# Supplementary material for: The Mechanism of the Channel Opening in Channelrhodopsin-2: A Molecular Dynamics Simulation
Source: Int J Mol Sci. 2023 Mar 16;24(6):5667. doi: 10.3390/ijms24065667 (PMC10057421; doi:10.3390/ijms24065667)
Supplement: Supplementary file 1 [file ijms-24-05667-s001.zip › ijms-2235680-supplementary.pdf]

Supplementary Information

The Mechanism of the Channel Opening in Channelrhodopsin-2: A Molecular Dynamics Simulation

Qi Xin, Shuai Yuan*, and Wenying Zhang*

Table S1 The protonation state of key residues of each intermediate. The “pro” and “depro” are abbreviations of protonation and deprotonation states respectively.

	D470	P500	P390-early	P390-late	P520
E90	pro	pro	pro	depro	depro
D156	pro	pro	pro	pro	depro
E123	depro	depro	pro	pro	pro
D253	depro	depro	depro	depro	depro
RSB	pro	pro	depro	depro	pro

Table S2 The pKa value of several key residues of each intermediate.

	D470	P500	P390-early	P390-late	P520
E90	6.43	6.98	10.52	3.00	3.26
E123	3.46	2.57	6.68	10.13	9.83
D156	7.38	6.34	6.15	6.70	5.87
D253	0.08	1.55	4.26	6.96	7.08

Table S3 Theoretical predicted λ_{max} of each intermediate at CAM-B3LYP/6-311++G(d,p) level.

Intermediates	D470	P500	P390-early	P390-late	P520
λ_{max} / nm	463.67	498.17	369.48	302.67	510.58

Table S4 The results of cluster analysis of ChR2 in D470, P500, P390-early, P390-late and P520 trajectories. All the conformations used in the paper are the optimal structures in the first column.

	Cluster 1 (%)	Cluster 2 (%)	Cluster 3 (%)
D470	52.1	22.6	20
P500	48.3	34.9	7.3
P390-early	49.1	16.4	16.2
P390-late	44.7	31.1	21.1
P520	42.9	33.1	20.2

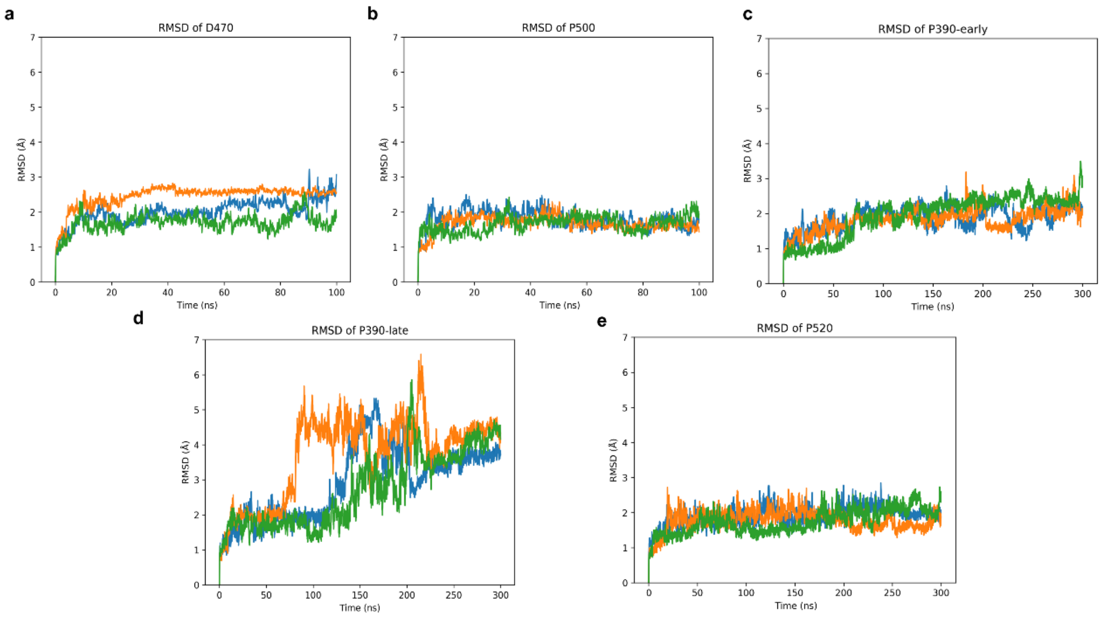

Figure S1 The RMSDs of (a) D470, (b) P500, (c) P390-early, (d) P390-late and (e) P520 intermediates in the photocycle. Each intermediate was run in three independent trajectories.

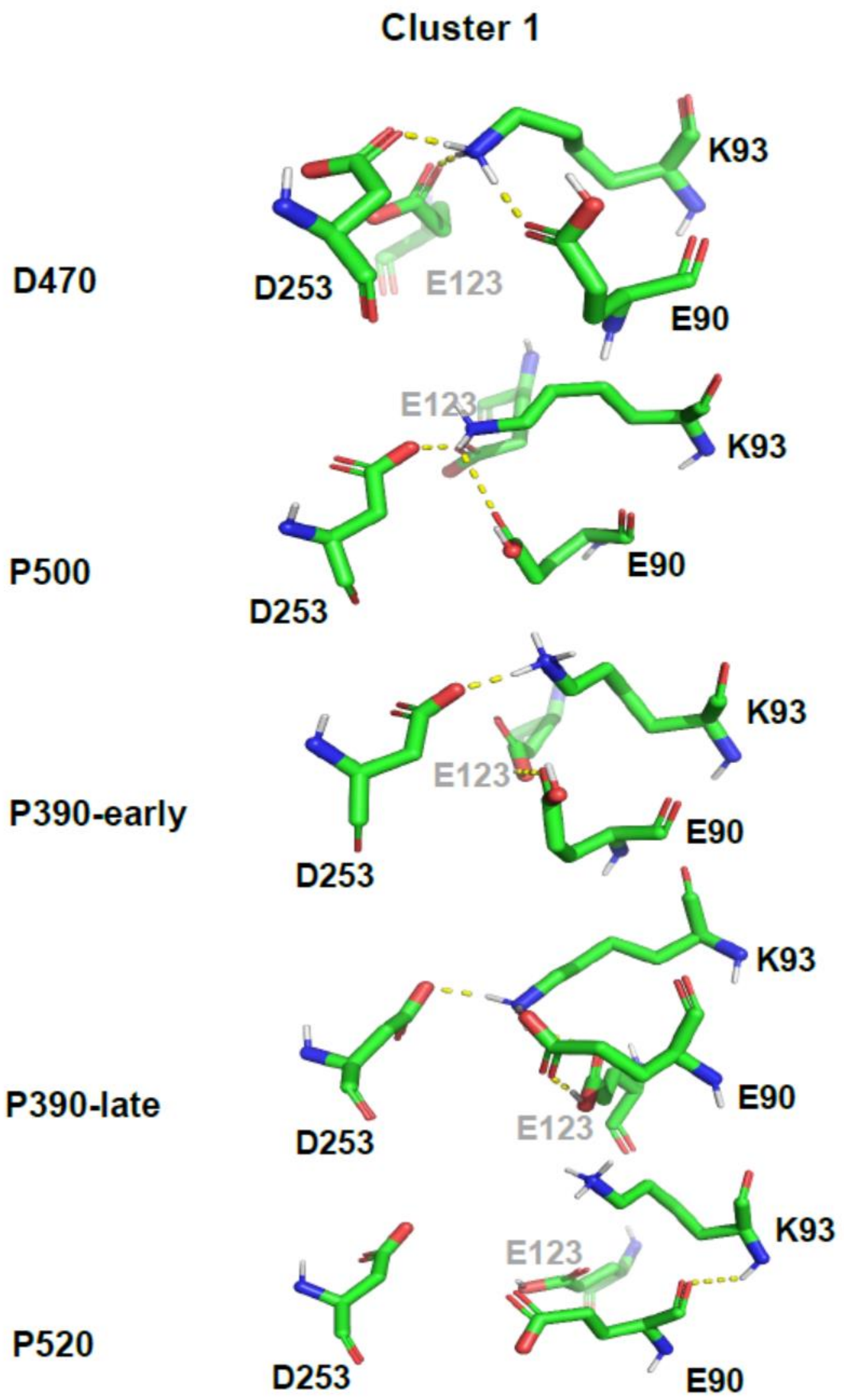

Figure S2 The CG structure of the cluster analysis of ChR2 in D470, P500, P390-early, P390-late and P520 states. The CG gradually opens as the photocycle occurs. All the conformations above are the optimal structures in the first column of Table S4 (Cluster 1).

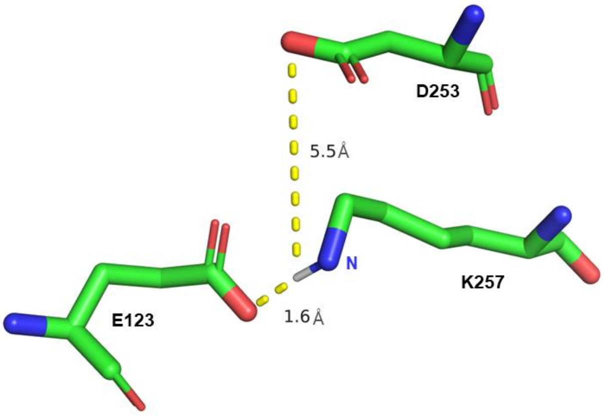

Figure S3 The distances between Schiff base and E123 and D253 in P500 intermediate. The distance between Schiff base and E123 is much shorter than that to D253 in our simulations, indicating the E123 is more likely to be a proton acceptor.

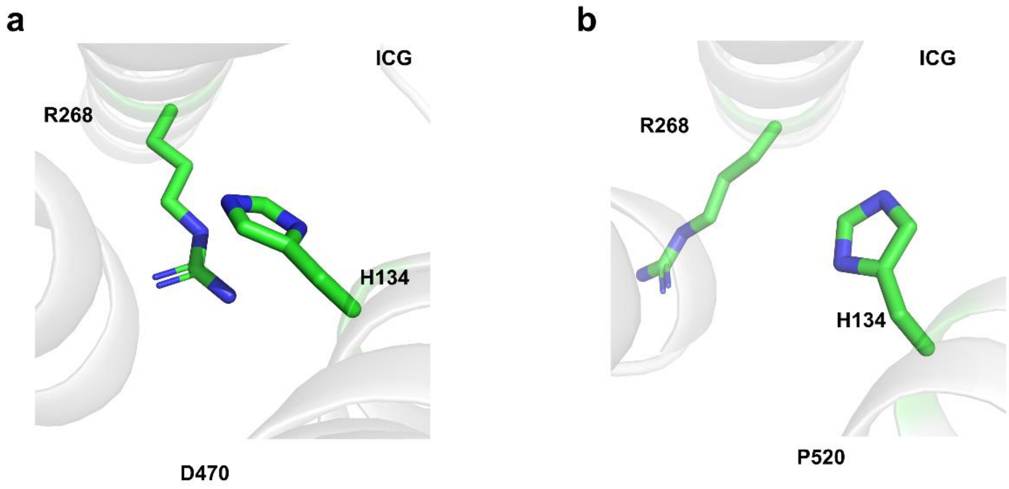

Figure S4 Local conformations of ICG of (a) D470 and (b) P520 intermediates. Compared to D470, the location of the side chain of R268 in P520 state is far from ICG, suggesting the ICG of P520 state is more open.

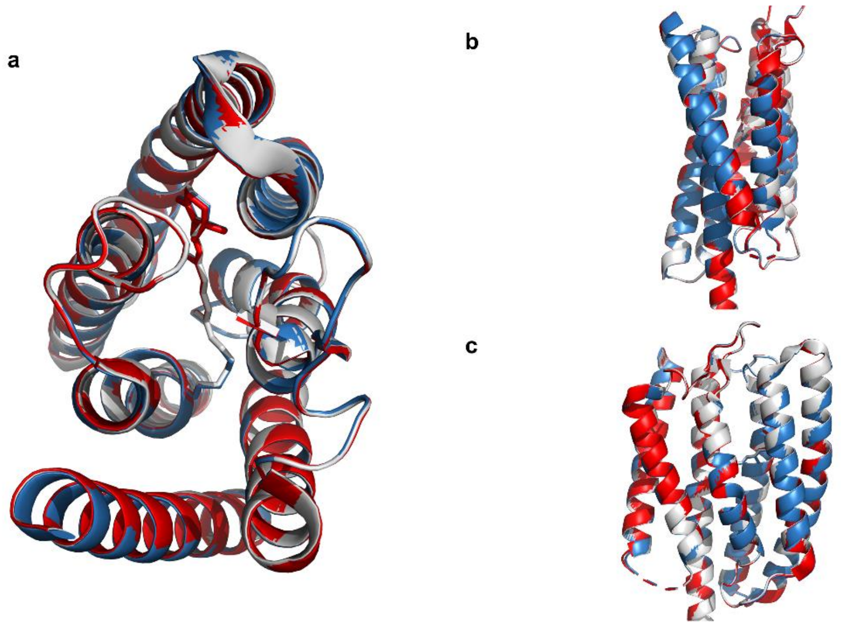

Figure S5 The aligned C1C2 crystal structures at 250μs (red), 1ms (blue) and 4ms (grey). (a) Top view (b) Side view (c) Front view

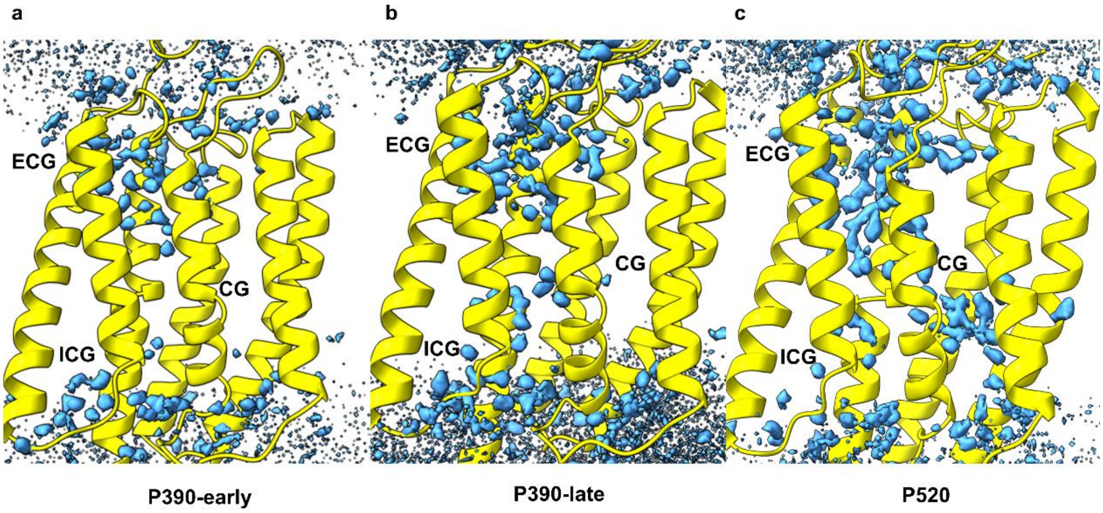

Figure S6 The water density distributions in (a) P390-early, (b) P390-late and (c) P520 intermediates.
